# L‐type voltage‐gated calcium channels in stem cells and tissue engineering

**DOI:** 10.1111/cpr.12623

**Published:** 2019-05-21

**Authors:** Yi‐zhou Tan, Dong‐dong Fei, Xiao‐ning He, Ji‐min Dai, Rong‐chen Xu, Xin‐yue Xu, Jun‐jie Wu, Bei Li

**Affiliations:** ^1^ Department of Periodontology, State Key Laboratory of Military Stomatology & National Clinical Research Center for Oral Diseases & Shaanxi Engineering Research Center for Dental Materials and Advanced Manufacture, School of Stomatology The Fourth Military Medical University Xi’an China; ^2^ State Key Laboratory of Military Stomatology & National Clinical Research Center for Oral Diseases & Shaanxi Key Laboratory of Oral Diseases, Center for Tissue Engineering Fourth Military Medical University Xi’an China; ^3^ Doctoral students of eight-year program The Fourth Military Medical University Xi’an China; ^4^ Department of Orthodontics, State Key Laboratory of Military Stomatology & National Clinical Research Center for Oral Diseases & Shaanxi Clinical Research Center for Oral Diseases, School of Stomatology The Fourth Military Medical University Xi’an China

**Keywords:** L‐type voltage‐gated calcium ion channels, myogenesis, neurogenesis, osteogenesis, stem cells, tissue engineering

## Abstract

L‐type voltage‐gated calcium ion channels (L‐VGCCs) have been demonstrated to be the mediator of several significant intracellular activities in excitable cells, such as neurons, chromaffin cells and myocytes. Recently, an increasing number of studies have investigated the function of L‐VGCCs in non‐excitable cells, particularly stem cells. However, there appear to be no systematic reviews of the relationship between L‐VGCCs and stem cells, and filling this gap is prescient considering the contribution of L‐VGCCs to the proliferation and differentiation of several types of stem cells. This review will discuss the possible involvement of L‐VGCCs in stem cells, mainly focusing on osteogenesis mediated by mesenchymal stem cells (MSCs) from different tissues and neurogenesis mediated by neural stem/progenitor cells (NSCs). Additionally, advanced applications that use these channels as the target for tissue engineering, which may offer the hope of tissue regeneration in the future, will also be explored.

## 1 INTRODUCTION

**Figure 1:**
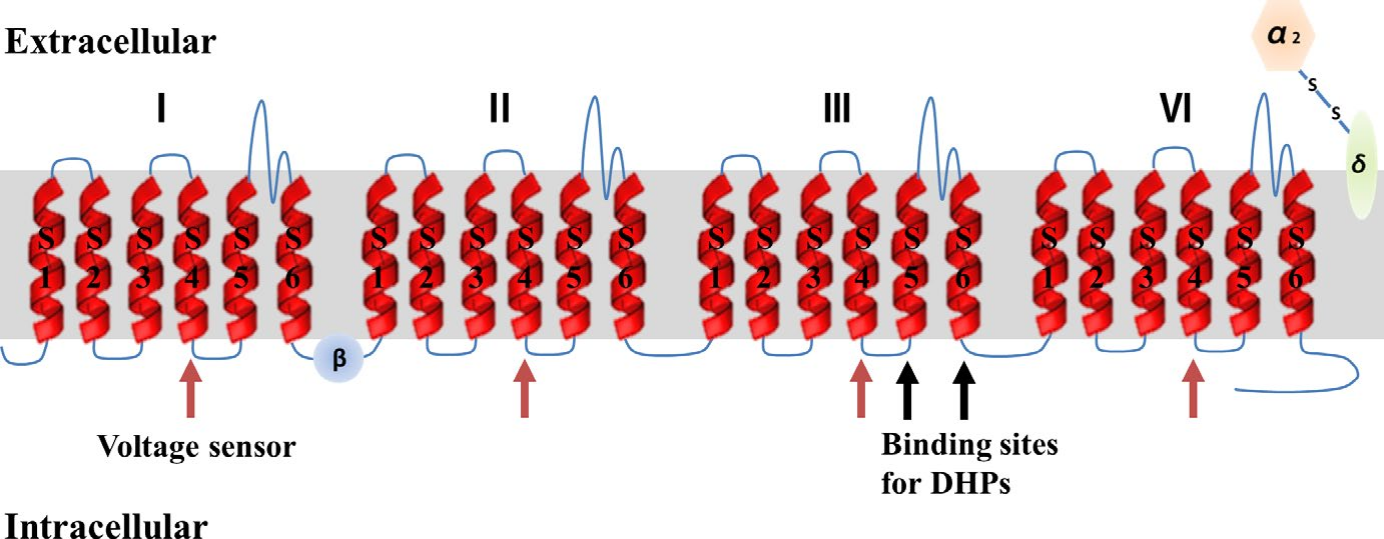
Topology of a voltage‐gated calcium channel subunit. L‐VGCCs are formed of subunits encompassing the pore‐forming α1 subunit, auxiliary subunits α2/δ, β subunit and γ subunit (not shown). The α1 subunit consists of four homologous repeats, each with six membrane‐spanning helices. The S4 helix in each repeat can serve as voltage sensor and the S5‐S6 helices in Repeat III are binding sites for L‐VGCCs blockers, especially for dihydropyridines (DHPs). The α2/δ subunit comprises an α2 subunit with an extracellular protrusion and a δ subunit buried within the cell membrane. The β subunit is the cytoplasmic protein containing an SH3 domain and a guanylate kinase‐like domain. The γ subunit can be absent, especially in heart tissue, which is not shown in Figure 1. L‐VGCCs, L‐type voltage‐gated calcium ion channels

Voltage‐gated calcium channels (VGCCs) are heteromeric membrane protein complexes characterized by depolarization‐induced calcium entry, which render the membrane highly permeable for Ca^2+^ ions (Figure 1). Based on their electrophysiological properties, VGCCs can be divided into low‐ and high‐voltage activated channels. The L‐type voltage‐gated calcium channels (L‐VGCCs), a major route of calcium influx, is a part of the high‐voltage activated family.^1^ They were named “L” for their long‐lasting inward currents during the depolarization process as studied in neurons and cardiac myocytes, and they are sensitive to 1,4‐dihydropyridines. The Ca^2+^ current mediated by L‐VGCCs can be stimulated by Bay K 8644 and FPL 64176, or blocked by nifedipine and nimodipine.^2^, ^3^ Furthermore, studies have demonstrated that calmodulin (CaM)‐dependent protein kinase II (CaMKII) is required for the basal activity of L‐VGCCs and the transduction of L‐VGCCs‐mediated signals into the nucleus.^4^, ^5^ Structurally, L‐VGCCs are composed of several different subunits, encompassing the main pore‐forming α1 subunit, auxiliary subunits α2/δ, β and γ.^6^ The α1 subunit, which contains four homologous repeats, determines the main biophysical and pharmacological properties of the channels including voltage sensing, ion permeability and drug binding. Each of the repeats is composed of six membrane‐spanning helices.^7^ Specifically, The S4 helix in each repeat can serve as voltage sensor and the S5‐S6 helices in Repeat III are binding sites for L‐VGCCs blockers, especially for dihydropyridines (DHPs). The α2δ subunit protrudes far into the extracellular space and influences the voltage‐dependent and kinetic properties of the calcium currents.^8^ It also takes part in cell attachment of skeletal myocytes and synaptogenesis in neurons.^5^ The β subunit is a cytoplasmic protein containing a conserved Src homology 3 (SH3) domain and a guanylate kinase‐like domain. It binds to the cytoplasmic linker between domains I and II of the α1 subunit via the guanylate kinase domain and facilitates trafficking of calcium channel complexes by preventing E3 ubiquitin ligase‐induced proteasomal degradation of the α1 subunit.^6^, ^9^ The γ subunit was purified from skeletal muscle, but it can be absent, especially in heart tissue. Compared with other auxiliary subunits, its role is limited and defined as a transmembrane AMPA‐glutamate receptor modifying protein.^6^ All these subunits can undergo alternative splicing, a process that enables a single gene to code for multiple proteins by rearranging the pattern of introns and exons. This process is a key mechanism for the regulation of pharmacological properties of L‐VGCCs, generating a variety of unique splice isoforms with distinct cell and tissue distribution, with significance for specific physiological and pathological processes.^10^, ^11^

According to differences of the α1 subunits, L‐VGCCs have four subtypes, ranging from Ca_v_1.1 to Ca_v_1.4, which are distributed widely among different tissues and cells, with distinct pharmacological and biophysical properties to support specific cellular tasks.^6^, ^12^ Calcium influx through these channels can serve as a second messenger of electrical signalling, initiating intracellular events such as membrane depolarization, secretion, synaptic transmission and gene expression.^6^, ^13^ Concretely, Ca_v_1.1, which is mainly present in skeletal muscle and expressed at low levels in other tissues, can serve as the voltage sensor mediating the process of excitation‐contraction coupling.^14^ Ca_v_1.2 is widely expressed in various tissues including smooth muscle,^15^, ^16^ bone,^17^ brain^18^, ^19^ and heart.^20^ In cardiac tissues, Ca_v_1.2 is indispensable for calcium ions to enter the cardiac cells and initiate cardiac excitation‐contraction coupling during the plateau phase of the action potential. Additionally, Ca_v_1.2 is also expressed in most types of neurons, where it activates calcium‐dependent enzymes and initiates calcium‐dependent gene transcription.^7^ The distribution pattern of Ca_v_1.3 is similar to that of Ca_v_1.2.^7^ Recently, Ca_v_1.3 has been shown to initiate pace‐making in dopaminergic (DA) neurons in the substantia nigra pars compacta (SNpc)^21^ and the sinoatrial node (SAN).^22^ The calcium currents associated with Ca_v_1.3 regulate the secretion of catecholamine from chromaffin cells^23^ and are also essential for the development of normal immature inner hair cells (IHC).^24^ Distinct from the wide distribution of Ca_v_1.2 and Ca_v_1.3, Ca_v_1.4 appears to be restricted to the retina,^25^, ^26^ where it is crucially important for the release of neurotransmitters.^27^ Due to the distribution and function of described subtypes, the structural aberrations within their pore‐forming α1 subunits can lead to a number of disorders,^7^, ^13^, ^28^ including hypokalemic periodic paralysis and malignant hyperthermia sensitivity (Ca_v_1.1), Timothy syndrome (a glycine‐to‐arginine mutation at position 406 in Ca_v_1.2), SAN dysfunction and deafness syndrome (Ca_v_1.3), and incomplete congenital stationary night blindness (Ca_v_1.4).

**Figure 2:**
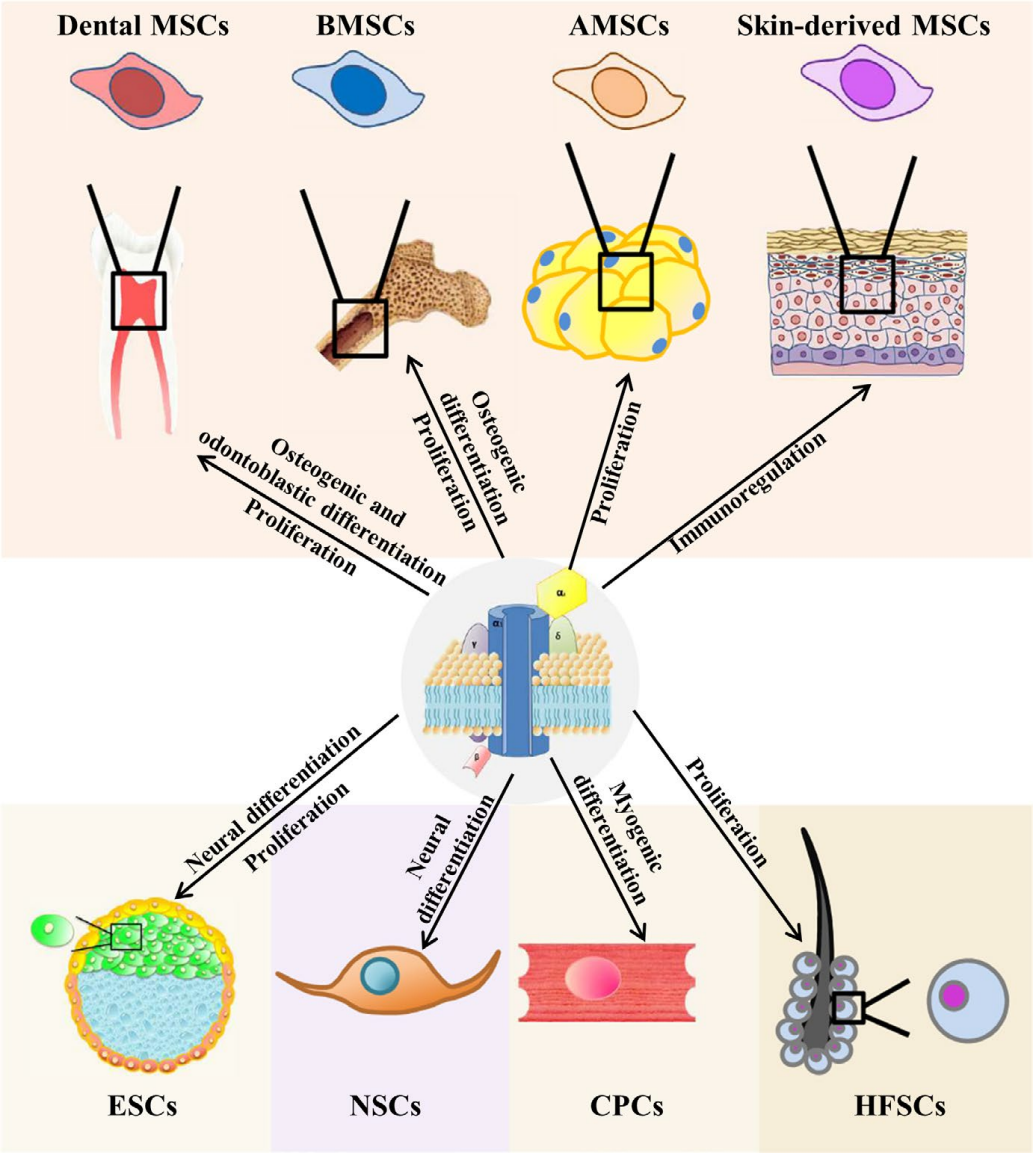
Functional regulation of various stem cell types by L‐VGCCs. L‐VGCCs are involved in the functional regulation of various stem cell types, including MSCs from bone marrow, oral cavity, adipose and skin tissues, as well as other stem cells, such as ESCs, NSCs, CPCs and HFSCs. The corresponding regulatory mechanisms influence cell proliferation and multipotent differentiation, such as osteogenic, neurogenic and myogenic differentiation. The γ subunit can be absent in cardiac and neuronal calcium channels. AMSCs, adipose‐derived mesenchymal stem cells; CPCs, cardiac progenitor cells; ESCs, embryonic stem cells; HFSCs, hair follicle stem cells; L‐VGCCs, L‐type voltage‐gated calcium ion channels; NSCs, neural stem/progenitor cells

While there is ample evidence supporting the vital function of L‐VGCCs in excitable cells including neurons,^29^ chromaffin cells^30^ and myocytes,^31^, ^32^ this mediation in non‐excitable cells such as stem cells has not been described firmly. However, recent studies have found that L‐VGCCs also contribute to the proliferation and differentiation of several kinds of stem cells. This review will discuss the possible involvement of L‐VGCCs in the function of stem cells isolated from different accessible sources (Figure 2), especially as it pertains to the osteogenic differentiation of MSCs and neurogenic differentiation of NSCs. Moreover, considering that the effects of L‐VGCCs on stem cells offer the hope of tissue regeneration in the future, we will also discuss the potential applications of L‐VGCCs in tissue engineering.

## 2 L‐VGCCS AND MSCS

Mesenchymal stem cells are multipotent cells that have a wide distribution in several tissues including bone marrow, adipose and dental tissues, and have the ability to differentiate into a large number of cell types including osteoblasts and adipocytes, as well as chondrocytes and neurons.^33^ Studies focusing on regenerative medicine have shed light on the capacity of MSCs for proliferation, multipotent differentiation and immunoregulation. Since the existence of L‐VGCCs has been detected in a wide range of MSCs from different species and tissues, we will discuss the important roles of L‐VGCCs in MSCs derived from different tissue sources, especially from bone marrow and oral tissue sources, and the functional regulation of these MSCs. The functional regulation of MSCs from different sources by L‐VGCCs was shown in Table 1.

**Table 1:** Functional regulation of MSCs from different sources by L‐type voltage‐gated calcium ion channels (L‐VGCCs)

Cell type	Source	Compound	Function	Mechanism	Reference
BMSCs	Rat	Nifedipine	Proliferation↓	╲	38
BMSCs	Rat	Nifedipine, Ca_v_1.2 siRNA	Osteogenic differentiation↓	Ca_v_1.2	38
BMSCs	Human	Nifedipine	Osteogenic differentiation↓	BMP‐2	39
BMSCs	Mouse	Benidipine	Osteogenic differentiation↑	Wnt/β‐catenin	40
DPSCs	Rat	Nimodipine Ca_v_1.2 shRNA	Odontoblastic differentiation↓	Ca_v_1.2	42
DPSCs	Human	Nifedipine	Odontoblastic differentiation↓	ERK1/2	43
SCAPs	Rat	Ca_v_1.2 shRNA	Odontoblastic and osteogenic differentiation↓	Ca_v_1.2	46
PDLSCs	Human	Nifedipine	Osteogenic differentiation↓	╲	47
AMSCs	Human	╲	Proliferation	Ca_v_1.1	50
S‐MSCs	Mouse	╲	Immunosuppression	╲	52

Abbreviations: AMSCs, adipose‐derived mesenchymal stem cells; BMSCs, bone marrow‐derived mesenchymal stem cells; DPSCs, dental pulp stem cells; PDLSCs, periodontal ligament stem cells; SCAPs, stem cells from the apical papilla; S‐MSCs, skin‐derived MSCs.

### 2.1 L‐VGCCs and bone marrow‐derived mesenchymal stem cells

Bone marrow‐derived mesenchymal stem cells (BMSCs) are acquired mainly from bone marrow, where they account for <0.0001% of all cells. Considering the advantages of easy accessibility, multipotent differentiation and low immunogenicity, BMSCs have been widely studied and hold great promise for treating a variety of immune‐mediated diseases and/or tissue defects.^34^ The patch‐clamp analysis demonstrated that functional L‐VGCCs are expressed in about 15% of human undifferentiated BMSCs,^35^, ^36^ which was similar to rat BMSCs,^37^ whereby calcium entry through L‐VGCCs has a significant impact on BMSCs properties. One study investigated the role of L‐VGCCs in the proliferation of rat BMSCs and found that blockade of L‐VGCCs by nifedipine had suppressed their proliferation.^38^ In terms of osteogenic differentiation, there is ample evidence for the involvement of L‐VGCCs. Bone morphogenetic protein 2 (BMP‐2) plays an important role in inducing osteoblast differentiation. Barradas et al^39^ tested the expression of BMP‐2 in human BMSCs after stimulation by nifedipine and observed significantly downregulated expression of BMP‐2, implying that L‐VGCCs could regulate osteogenic differentiation through influencing BMP‐2 expression. Li Wen et al^38^ demonstrated that inhibition of L‐VGCCs can suppress the osteogenic differentiation of rat BMSCs. However, data from mouse BMSCs were inconsistent with these results from rat BMSCs. For example, Ma et al^40^ also tested the role of L‐VGCCs in the osteogenic differentiation in an ovariectomized (OVX) mouse model and found that the L‐type calcium channel blocker benidipine promoted the differentiation of BMSCs into osteoblasts and mitigated the symptoms of osteoporosis in OVX mice by upregulating the Wnt/β‐catenin pathway. Such different outcomes may be caused by the non‐specificity of nifedipine and benidipine, as well as different L‐VGCCs subtypes present between cells from different species and tissues.

### 2.2 L‐VGCCs and dental MSCs

Dental tissues are accessible resources that provide an abundant reservoir of MSCs, such as dental pulp stem cells (DPSCs), stem cells from exfoliated deciduous teeth (SHED), stem cells from the apical papilla (SCAPs) and periodontal ligament stem cells (PDLSCs). To date, many studies have investigated the involvement of L‐VGCCs in regulating the functions of dental MSCs. DPSCs are derived from dental pulp and possess the capacity for multi‐lineage differentiation towards bone, cartilage and fat.^41^ Studies have been indicated that the L‐VGCCs blocker nimodipine inhibits the odontogenic differentiation of DPSCs from rats, and knock‐down of Ca_v_1.2 can inhibit the differentiation of rDPSCs.^42^ Furthermore, Li et al found that L‐VGCCs promote the expression of dentin sialophosphoprotein (DSPP) and odontoblastic differentiation of DPSCs from humans.^43^ They also found that the enhanced odontoblastic differentiation of DPSCs was regulated by L‐VGCCs through the Smad1/5/8 and Erk1/2 pathways.^43^ However, recent experiments by Mizumachi et al suggest that the calcium‐sensing receptor‐ERK signalling rather than L‐VGCCs is responsible for the observed effect on odontogenic differentiation of human dental pulp cells, indicating the involvement of different pathways in each cell type.^44^ Recently, a novel population of MSCs was discovered in the apical papilla, the SCAPs. These stem cells were demonstrated to have a stronger ability of odontogenic differentiation and continued root formation compared to DPSCs.^45^ In rat SCAPs, Gao et al confirmed that Ca_v_1.2 also plays significant roles in the odontoblastic differentiation. Using Ca_v_1.2 shRNA to knock‐down Ca_v_1.2 in SCAPs, they found that the formation of calcium nodule, a product of late stages of osteogenic differentiation that can reflect the ability of stem cells to differentiate into osteoblasts, was suppressed.^46^ In human PDLSC lines (cell lines 1‐17), elevated extracellular calcium was found to lead to the enhanced proliferation and osteogenic differentiation, while the L‐VGCCs inhibitor nifedipine was able to suppress extracellular calcium‐mediated osteogenic differentiation.^47^

### 2.3 L‐VGCCs and MSCs from other tissues

In addition to the bone marrow, MSCs also exist in many other tissues, including adipose and skin tissues. The patch‐clamp analysis demonstrated that human adipose‐derived mesenchymal stem cells (AMSCs) possess functional VGCCs, although they account for <1%.^48^ However, analysis of cytoplasmic Ca^2+^ concentration evoked by ATP, high K^+^ solution, GABA and caffeine demonstrated that functional VGCCs are absent in AMSCs isolated from rats.^49^ The previous work on AMSCs from chronic kidney disease patients by Thi et al had indicated a possible association between Ca_v_1.1 and defective proliferation of AMSCs. AMSCs displayed a decreased proliferation capacity and increased apoptosis after treatment with indoxyl sulphate, a digestive intermediate product that can induce chronic kidney disease, along with significantly downregulated expression of Ca_v_1.1.^50^ L‐VGCCs are also closely related to immune responses. Generally speaking, blocking L‐VGCCs can lower the immune response, as DHPs can suppress the proliferation of immune cells.^12^ Besides, the combination of DHPs and cyclosporine A have been combined to be frequently used in transplantation patients.^51^ In skin‐derived MSCs, L‐VGCCs are involved in autocrine interleukin‐6‐mediated cell migration and immunosuppressive function through mediating contraction, a process that can help MSCs migration.^52^

## 3 L‐VGCCS AND EMBRYONIC STEM CELLS

Embryonic stem cells (ESCs) are isolated from the inner cell mass of the preimplantation embryo. During embryogenesis, elevated intracellular Ca^2+^ is needed for ESCs to differentiate towards a specific tissue fate.^53^ The expression of L‐VGCCs has been identified in ESCs from humans and mice. However, functional L‐VGCCs could be identified only in undifferentiated mouse ESCs and are absent in human ESCs, which was confirmed by analysis of cytoplasmic Ca^2+^ concentration evoked by ATP, high K^+^ solution, GABA and caffeine.^49^ Huang et al carried out experiments on human ESC lines H7 and H9, indicating that these might be voltage‐insensitive cells, as depolarization‐induced Ca^2+^ entry by a high K^+^ solution did not change the intracellular free Ca^2+^ concentration.^54^ L‐VGCCs have been demonstrated to regulate the proliferation of mouse ESCs (mESCs).^55^ In addition, neural differentiation of mESCs is also strongly enhanced by L‐VGCCs.^56^, ^57^ Experiments performed by Lepski et al showed that the application of IBMX (3‐isobutyl‐1‐methylxanthine), which caused a prominent increase of L‐type Ca^2+^ currents, significantly promoted neuronal functional maturation, and this process could be inhibited by the L‐VGCCs antagonist nifedipine. Mechanistically, they discovered complete inhibition of IBMX‐induced CREB phosphorylation (cAMP‐response‐element‐binding protein) by nifedipine, which indicates that L‐VGCCs‐mediated maturation of NSCs depends on enhanced intracellular cAMP levels.^58^ In addition, Yu et al found that intracellular Ca^2+^ signalling was significantly inhibited and neurogenesis was also decreased in mESCs deficient in the Ca^2+^ release channel type 2 ryanodine receptors (RyR2). At the same time, the activation of L‐type Ca^2+ ^channels or ryanodine receptors promoted neuronal differentiation in RyR2^+/+^ cells but not in RyR2^−/−^ deficient mESCs. Thus, cooperation of L‐VGCCs and RyR2 is indispensable for neural differentiation of mESCs.^57^, ^59^

## 4 L‐VGCCS AND NSCS

Neurogenesis is a process with several stages such as proliferation, fate determination, selective death/survival and maturation, in which neurons or glial cells are generated from NSCs and other neural progenitor cells. NSCs play an essential role in neural development because of their ability for pluripotent differentiation and are most active during pre‐natal neurosystem development. By contrast, adult NSCs, which are considered to be limited in their ability of differentiation,^60^ are found in only two neurogenic regions in the adult brain. One is the subventricular zone of the lateral ventricles (SVZ) where NSCs generate cells that migrate into the olfactory bulb, and the other is the subgranular zone (SGZ) of the dentate gyrus (DG) where new granule cells become integrated into the local neuronal network.^61^, ^62^

Several studies suggest that the L‐type calcium current has a strong correlation with the differentiation of NSCs. Ca_v_1.2 and Ca_v_1.3 are the predominant L‐VGCCs isoforms expressed in several regions of the central nervous system (CNS), including the cerebral cortex, amygdala, cerebellum and hippocampus. They are deeply involved in the physiological behaviours of the CNS such as cognition and memory formation.^63^ A further study^64^ has indicated that L‐VGCCs might be involved in the differentiation of neural progenitor cells towards neurons, enhancing survival of immature neurons and facilitating maturation. Furthermore, the L‐VGCCs‐dependent regulation targets the later stages of neurogenesis. To be specific, L‐VGCCs activation and subsequent Ca^2+^ influx facilitate the survival and maturation of immature neurons.^64^ Another study^65^ has also found that specifically Ca_v_1.2 is only expressed in cells undergoing the final stages of neuronal differentiation. The neurogenesis controlled by L‐VGCCs can be enhanced by the L‐channel activator Bay K 8644, as well as by an agonist of Ca_v_1.2/Ca_v_1.3, FPL 64176.^64^, ^66^ Ca_v_1.3 knock‐down rats produced using adeno‐associated virus (AAV) or retrovirus‐based technologies showed impaired dendritic or axonal formation in vivo.^63^

To further study the mechanism of the regulation of neurogenic activities by L‐VGCCs, researchers investigated the involvement of Ca^2+^‐dependent regulatory genes in differentiation, such as BETA2/NeuroD1,^64^ which are essential to neuronal survival and/or maturation in many neuronal tissues, including the dentate hippocampus, the cerebellum and the olfactory bulb,^67^, ^68^ and the research showed that BETA2/NeuroD1 were involved in L‐VGCCs‐mediated regulation. Moreover, it has been reported that activation of the GABA_B_ receptor can increase Ca_v_1.3 expression in NSCs,^69^ suggesting that GABA receptors may be a part of a signalling pathway for stem cell maintenance. The depolarization mediated by glutamate and GABA_A_ receptors would facilitate the Ca^2+^ influx through L‐type Ca^2+^ channels.^64^ However, the role of GABA receptors in the regulation of L‐VGCCs is controversial, since the evidence indicating that GABA receptors may be involved in the maintenance and activation of hippocampal NSCs is inconsistent^70^.

## 5 L‐VGCCS AND OTHER STEM CELLS

Cardiac progenitor cells (CPCs), which are mainly located in the myocardium, express the cardiogenic genes.^71^ They have the capability to differentiate into mature cardiomyocytes after stimulation by signal molecules. Researchers have shown the existence of several functional molecular elements of calcium signalling in CPCs, among which L‐VGCCs are crucial for proliferation and differentiation. Experiments performed by Hotchkiss et al showed that inhibition of L‐VGCCs in CPCs can suppress their proliferation and impair the cardiomyocyte contractile apparatus.^72^ L‐VGCCs are also engaged in functional regulation of hair follicle stem cells (HFSCs). The L‐VGCCs present in the bulge of the hair follicle are responsible for hair growth. HFSCs control hair follicle cycling including active growth, involution and quiescence.^73^ In spite of their lack of voltage‐dependent calcium currents, Ca_v_1.2 is expressed by HFSCs, where it inhibits quiescence and contributes to tissue regeneration in a calcium‐independent signal by regulating the production of the follistatin‐like 1 protein, an antagonist of BMP signalling.^74^

## 6 POTENTIAL APPLICATIONS OF L‐VGCCS IN TISSUE ENGINEERING

**Figure 3:**
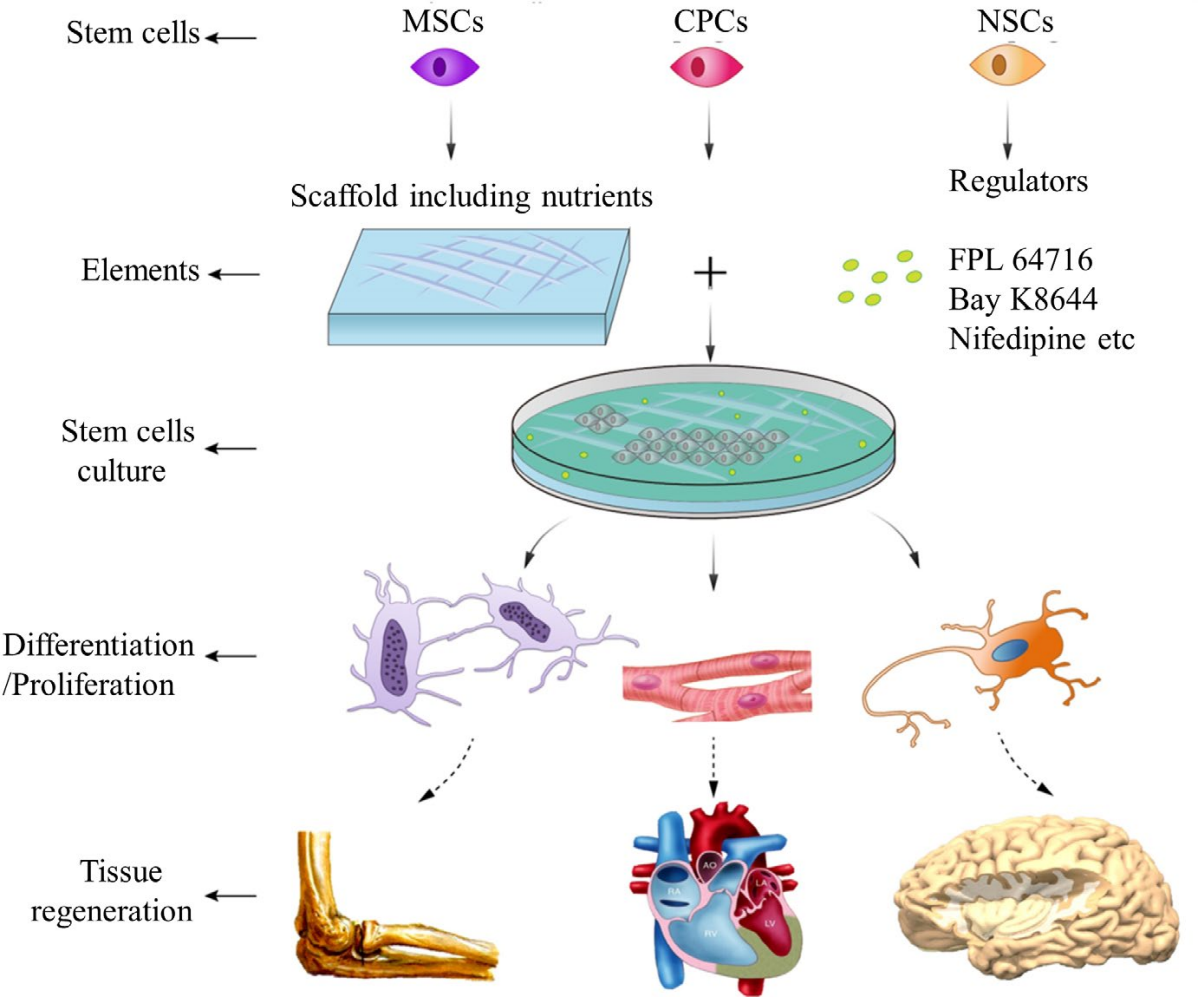
Potential application of L‐VGCCs in tissue regeneration. Biomolecules targeting L‐VGCCs are potential drugs for tissue regeneration. The application of scaffolds containing various stem cell types and regulators of L‐VGCCs is a new avenue for tissue regeneration. After management of L‐VGCCs to control the differentiation and/or proliferation of stem cells, tissue regeneration (MSCs for osteogenesis, NSCs for neurogenesis and CPCs for cardiogenesis) can be maximized by tissue engineering techniques. CPCs, cardiac progenitor cells; L‐VGCCs, L‐type voltage‐gated calcium ion channels; NSCs, neural stem/progenitor cells

Tissue engineering is an emerging interdisciplinary field involving the use of an interactive triad of scaffolds, signalling molecules, engineering and cells, focusing on producing functional replacement tissues and creating favourable conditions for tissue regeneration.^75^ The latest developments in tissue engineering have provided new perspectives for the regeneration and replacement of defective tissues, such as bone and heart tissue.^76^ Signalling molecules, which are vital for all tissue engineering strategies, can promote spatiotemporal signalling cascades to maximize the functionality of engineered tissues.^77^ Given that L‐VGCCs are of significance for the osteogenic, myogenic and neural differentiation of several stem cell types, we will focus on their potential applications in the tissue engineering of bone, heart and neurons for organ repair and regeneration (Figure 3).

### 6.1 L‐VGCCs and bone tissue engineering

**Figure 4:**
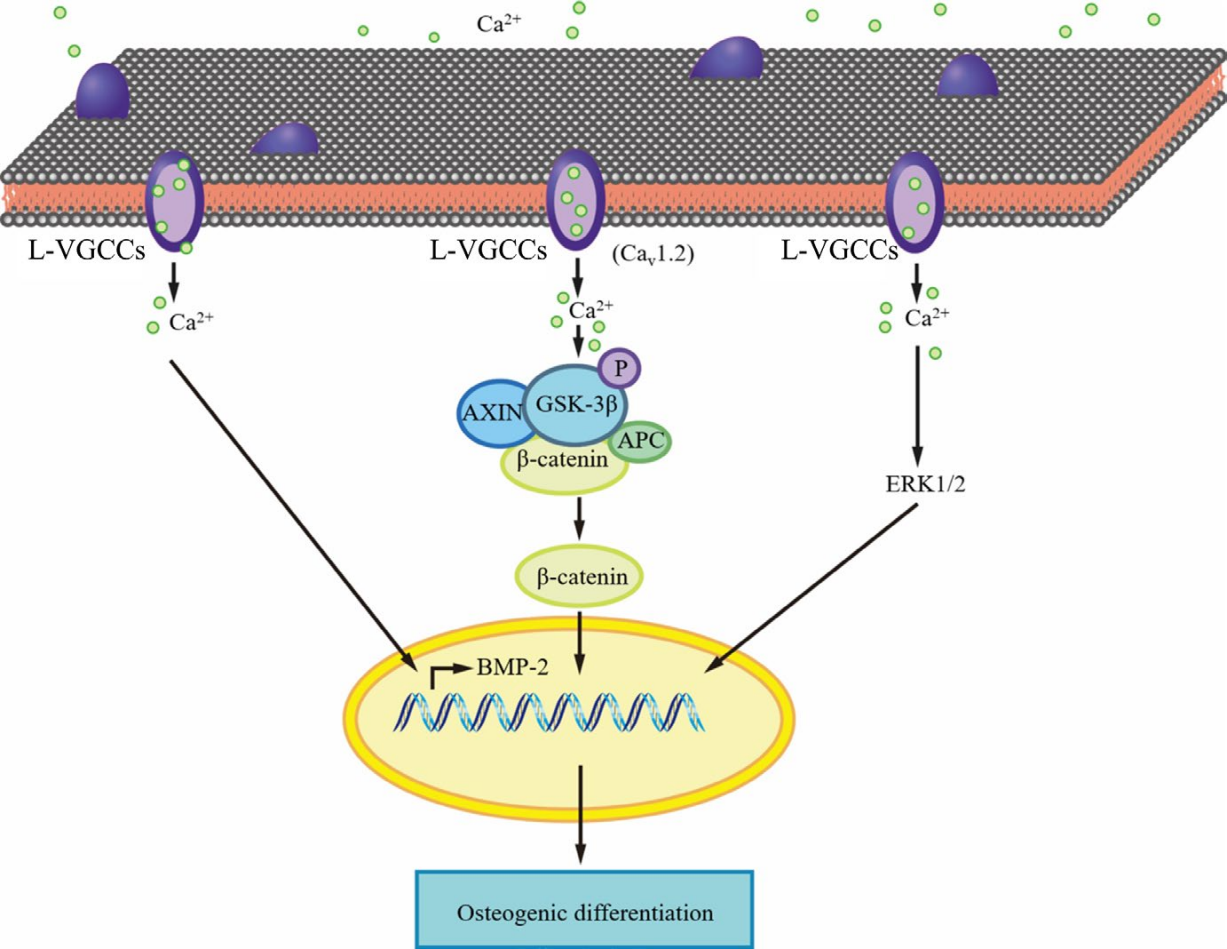
The role of L‐VGCCs in osteogenesis. L‐VGCCs have been implicated in regulating osteogenic differentiation of MSCs. There is significant evidence that Cav1.2 is associated with the regulation of osteogenic differentiation. The two signalling pathways, ERK and Wnt/β‐catenin, have been revealed to promote osteogenic differentiation mediated by L‐VGCCs. In addition, BMP‐2 is also involved in L‐VGCCs‐mediated osteogenesis. APC, adenomatous polyposis coli; AXIN, axis inhibition; ERK1/2, extracellular signal‐regulated kinase1/2; GSK‐3β, glycogen synthase kinase 3β; L‐VGCCs, L‐type voltage‐gated calcium ion channels; P, phosphate

Large bone defects caused by trauma, infections and other causes^78^ abrogate the bone's ability for spontaneous healing and consequently pose a great challenge for clinical treatment.^79^ Bone tissue engineering is a promising solution for treating such defects by providing a scaffold, combined with signalling molecules and cells, to guide the regeneration of osseous tissue.^80^ Application of scaffolds loaded with biological molecules that stimulate cell proliferation and osteogenic differentiation is one of the most investigated techniques for promoting the bone formation ability.^81^ Several biomolecules, such as bone morphogenetic proteins (BMPs), Wnt signalling molecules and transforming growth factor‐β (TGF‐β), have been applied in the regulation of osteogenesis to enhance bone healing.^79^, ^80^ Currently, some studies showed that MAPK and Wnt signalling pathways are involved in L‐VGCCs‐mediated osteogenesis. In addition, BMP‐2 was also reported downstream of L‐VGCCs. These studies support a proposed mechanism of L‐VGCCs‐mediated osteogenesis in MSCs that is summarized in Figure 4. We are also investigating the effects of the subtypes of L‐VGCCs on different signalling pathways. In view of the role of L‐VGCCs in osteogenic differentiation of several stem cell types, L‐VGCCs agonists such as Bay K8644 should be ideal candidates for bone tissue engineering. In fact, some studies have paved the way for translational application of L‐VGCCs agonists as potential biomolecules for bone regeneration. Diomede et al seeded human PDLSCs onto a porcine cortico‐cancellous scaffold which promoted osteogenic differentiation of human PDLSCs in vitro and osteointegration in vivo. Mechanistically, they found significant increases of calcium transients and gene expression of Ca_v_1.2 and α2D1 in undifferentiated human PDLSCs, suggesting that L‐VGCCs may be a potential target for periodontal tissue engineering.^82^ Other researchers also elucidated that L‐VGCCs are involved in in vitro osteogenic differentiation induced by calcium phosphate (CaP)‐bearing biomineralized scaffolds, as well as in in vivo bone tissue regeneration of human ESCs and MSCs.^83^ Thus, the application of L‐VGCCs agonists is a promising approach for bone tissue engineering.

### 6.2 L‐VGCCs and cardiomyogenesis

Heart failure, especially myocardial infarction, is one of the leading global health challenges that threaten the well‐being of more than thirty million people annually.^84^ Resident cardiomyocytes of patients with heart disease have a compromised proliferation and differentiation potential. Current strategies for treating heart dysfunction, such as organ transplantation, are limited due to immunological rejection and insufficient availability of donor organs.^85^ Tissue engineering has emerged as a promising therapeutic strategy for cardiac regeneration. To date, diverse cell sources have been adopted for repair and regeneration of the impaired myocardium in animal models, with a subset of them undergoing clinical assessment.^86^ Stem cell‐based therapy has been considered as an attractive option for treating defective myocardium.^87^, ^88^ Several stem cell types have been used in clinical trials for treating functional deficiency of the heart, such as CPCs. A modest improvement of cardiac performance was observed in several clinical trials, but the methodology is still inadequate, partly due to functional immaturity and electrical insufficiency.^83^ The addition of small‐molecule compounds in tissue engineering is favourable to structural and functional recovery of the treated hearts.^89^ However, this approach is complicated by the fact that heart regeneration is a process involving cell proliferation, differentiation and maturation.^90^ Adequate knowledge about the regulatory processes that are active during cardiomyogenesis is indispensable for using biomolecules with unique activity to target stem cell function.

Researchers have confirmed that calcium influx is an integral second messenger for the early steps of cardiomyocyte specification and commitment, leading to the differentiation of stem cells by modulating cardiac gene expression.^71^ Recent data also showed that Ca^2+^ regulates excitation‐contraction coupling, as well as the localization of cardiogenic transcription factors in CPCs.^72^ Calcium channels, and particularly the L‐VGCCs, regulate the early differentiation of ESCs into cardiomyocytes. Chan et al cultured mouse embryos with nifedipine or verapamil, which caused their hearts to develop an enlarged left ventricle and a long, thin outflow tract.^91^ In fully differentiated adult cardiomyocytes, Ca_v_1.2 is believed to be the predominant L‐VGCCs expressed in ventricular myocytes, whereas both Ca_v_1.2 and Ca_v_1.3 are expressed in atrial cells as well as sinoatrial and atrioventricular node cells.^92^ Ca^2+^ influx through Ca_v_1.2 channels triggers the intercellular release of Ca^2+^ from the sarcoplasmic reticulum through type‐2 ryanodine receptors (RyR2), which is essential for myocardial excitation‐contraction coupling activity, and loss‐of‐function mutations of L‐VGCCs can lead to cardiac failure.^93^, ^94^ Some scholars have investigated the links between L‐VGCCs and heart regeneration, which makes drugs targeting L‐VGCCs an attractive candidate for treating heart failure through tissue engineering techniques. Ma et al^95^ carried out experiments on induced pluripotent stem cells which indicated that calcium influx through L‐VGCCs is crucial for electrical stimulation‐mediated improvement of cardiac differentiation and cardiac function, with attenuated expansion of the infarction region.

### 6.3 L‐VGCCs and neurogenesis

Since adult NSCs are limited in differentiation and distribution, neurons are refractory to replication. The reprogramming or regeneration of human neural tissue^96^ is considered to be difficult to achieve. According to several studies, the predominant isoforms of L‐VGCCs in the CNS are Ca_v_1.2 and Ca_v_1.3, which are widely expressed in multiple brain regions such as the cerebral cortex, amygdala, cerebellum and hippocampus,^97^ as well as in adult neurogenic regions that are involved in basic neural activities such as membrane depolarization and message communication in the neural tissue. Researchers found a significant reduction in neurogenesis in the DG in the hippocampi of adult and middle‐aged Ca_v_1.3^−/−^ mice with severe impairments in hippocampus‐associated cognitive functions,^65^ which may suggest Ca_v_1.3 involves in neurogenesis and cognitive function formation, giving promising future for neurological recovery enhancement through L‐VGCCs.

Considering the indispensable role of L‐VGCCs in neurogenesis, researchers are striving to find ways to modulate neurogenic activities by targeting L‐VGCCs, with some success. The exposure to extremely low‐frequency (50‐Hz) electromagnetic fields (ELFEFs) was found to increase the expression and modulate the function of voltage‐gated Ca^2+^ channels, especially Ca_v_1.2 and Ca_v_1.3.^98^ Ultimately, larger‐amplitude Ca^2+^ influx and a higher percentage of responsive neurons results in increased proliferation and neural differentiation. The exposure can also stimulate CREB phosphorylation, which mediates the effects of intracellular Ca^2+^ signals on gene expression and is thus involved in neuronal differentiation and neurogenesis.^99^, ^100^ Stimulation by the vitamin E isomer δ‐tocopherol increases the expression of voltage‐dependent Ca^2+ ^channels, which in turn led to neural differentiation, as well morphological and functional maturation of neural stem cells.^101^

## 7 CONCLUSIONS

In this review, we discussed not only the important roles of L‐VGCCs in the functional regulation of several stem cell types, but also the potential application of drugs targeting L‐VGCCs in tissue engineering. As discussed above, L‐VGCCs partake in the regulation of osteogenic, myogenic and neural differentiation, which makes their agonists or antagonists highly promising candidates for the repair and regeneration of bone, heart and neural tissue.

Notwithstanding, there are still considerable challenges within this field. First, functional L‐VGCCs are the precondition for the efficacy of the drugs targeting them, but they are present only in a portion of stem cells. Secondly, the role of L‐VGCCs in osteogenic differentiation still remains controversial, partly due to the varying characteristics of different stem cell types. Thus, more basic research is needed before attempting the therapy targeting L‐VGCCs for bone regeneration. Thirdly, it has been reported that L‐VGCCs can be detrimental to possibly cause severe neurodegenerative diseases. For example, researchers found that increasing Ca^2+^ influx by the overexpression of L‐VGCCs modulated the processing of the beta amyloid (Aβ) precursor protein, leading to a positive feedback loop and ultimately the development of Alzheimer's disease.^102^, ^103^ Despite this, it is clear that selective application of L‐VGCCs activators or inhibitors based on their effect to distinct stem cell types provides a new avenue for tissue regeneration.

## CONFLICT OF INTEREST

The authors have declared that no competing interest exists.

## AUTHOR CONTRIBUTIONS

Yi‐zhou Tan, Dong‐dong Fei and Xiao‐ning He: Conception and design, manuscript writing; Ji‐min Dai, Rong‐chen Xu and Xin‐yue Xu: Manuscript writing; Jun‐jie Wu and Bei Li: Conception and design, financial support, final approval of manuscript.
